# U1 RNA Detected by Toll-Like Receptor 3 Plays a Role in the Pathogenesis of Pterygium

**DOI:** 10.1167/iovs.66.15.15

**Published:** 2025-12-03

**Authors:** Chun-Chieh Lai, Cheng-Jhe Wu, Sung-Huei Tseng, Sheng-Min Hsu, Yin-Ting Huang, Chi-Chang Shieh

**Affiliations:** 1 Department of Ophthalmology, National Cheng Kung University Hospital, College of Medicine, National Cheng Kung University, Tainan, Taiwan; 2 Department of Medicine, College of Medicine, National Cheng Kung University, Tainan, Taiwan; 3 Department of Pediatrics, National Cheng Kung University Hospital, College of Medicine, National Cheng Kung University, Tainan, Taiwan; 4 Institute of Clinical Medicine, College of Medicine, National Cheng Kung University, Tainan, Taiwan

**Keywords:** non-coding RNA, pterygium epithelial cells, toll-like receptor 3, NF-κB, UVB irradiation

## Abstract

**Purpose:**

Toll-like receptor 3 (TLR3) detects RNA from pterygium epithelial cells (PECs). We previously suggested that pterygium development may be linked to RNA released from abnormally growing PECs: U1 RNA released from ultraviolet B (UVB)-damaged cells may activate TLR3. This study investigated how U1 RNA, polyriboinosinic:polyribocytidylic acid (poly[I:C]), and PEC lysates affect the TLR3 signaling pathway in PECs and conjunctival epithelial cells (CECs).

**Methods:**

Human pterygium and ipsilateral pterygium-free conjunctiva from the same patients were used for cell culture and RNA-sequencing analysis. PECs and CECs were cultured, irradiated with UVB, and treated with poly(I:C), PEC lysates, or synthetic U1 RNA. TLR3 and toll/interleukin-1 receptor domain-containing adaptor-inducing interferon-β (TRIF) expression, phosphorylated nuclear factor-kappa B (NF-κB)/NF-κB ratio, IL-6, and IL-8 were evaluated using western blot, quantitative real-time PCR (qPCR), and enzyme-linked immunosorbent assay (ELISA). Cell proliferation was evaluated using water-soluble tetrazolium salt-1 assay.

**Results:**

After UVB irradiation, U1, U2, U4, and U6 RNA increased in PECs and CECs, and TLR3 expression increased in PECs. Western blot and qPCR results indicated an increase in TLR3, TRIF, and NF-κB expression in PECs and CECs treated with poly(I:C), UVB-irradiated PEC lysates, or synthetic U1 RNA compared to controls. However, RNase A inhibited this effect in UVB-irradiated PECs. ELISA showed that IL-6 and IL-8 increased in cell groups treated with poly(I:C), UVB-irradiated PEC lysates, or synthetic U1 RNA. Proliferation of PECs was also increased by poly(I:C).

**Conclusions:**

Several small noncoding RNAs, whose expression was induced by UVB irradiation, may be a possible novel therapeutic target for pterygium treatment through activation of the TLR3 signaling pathway.

Pterygium is a common corneal disorder in subtropical, tropical, and other regions of high levels of ultraviolet albedo, especially in people who have experienced prolonged exposure to ultraviolet radiation.^1^^,^^2^^,^^3^ Ultraviolet B (UVB) has been shown to induce pterygium, manifested as chronic inflammation, abnormal extracellular matrix accumulation, and tissue invasion.^1^^,^^4^ In our previous study, UVB irradiation increased Toll-like receptor-3 (TLR3) and phospho-nuclear factor-kappa B (NF-κB) levels in pterygium epithelial cells (PECs).^5^ In addition, UVB-treated PECs showed higher IL-6 and IL-8 production.^6^ In this study, we focused on the TLR3 pathway and downstream signaling molecules and cytokines, including TLR3, Toll/interleukin-1 receptor domain-containing adaptor-inducing interferon-β (TRIF), NF-κB, IL-6, and IL-8.

TLR3 is a double-stranded RNA (dsRNA)-sensing pattern-recognition receptor, whose activation is necessary for skin repair after UVB damage.^7^ In addition, TLR3 activates NF-κB by interacting with TRIF.^8^^,^^9^ UVB irradiation leads to the release of intracellular noncoding RNAs (ncRNAs) in human keratinocytes, and their cell lysates further stimulate IL-6 and TNF-α production in non-irradiated keratinocytes.^7^^,^^10^^,^^11^ Furthermore, the TLR3 ligand polyriboinosinic:polyribocytidylic acid (poly[I:C]) and ncRNA U1 have been found to induce skin repair genes dependent on TLR3 as a response to UVB damage.^7^^,^^10^ Therefore, we investigated how poly(I:C) and U1 RNA influenced the TLR3 signaling pathway in conjunctival epithelial cells (CECs) and PECs.

We previously demonstrated that the levels of expression of TLR3, p63, and NF-κB were higher in pterygium than in ipsilateral pterygium-free conjunctiva.^5^ Furthermore, UVB radiation induced the upregulation of TLR3 and nuclear translocation of NF-κB in PECs. In contrast, the knockdown of TLR3 suppressed UVB-induced phosphorylation of NF-κB in PECs.^5^

We hypothesized that TLR3 contributes significantly to UV-related pterygium by recognizing self-noncoding RNA danger-associated molecular patterns (DAMPs) released from UV-damaged necrotic cells. TLR3 activation triggers NF-κB translocation, leading to pterygial cell proliferation through p63 production. To test this RNA-mediated mechanism, we evaluated poly(I:C), UVB-irradiated PEC lysates, and U1 RNA as potential TLR3 pathway activators.

## Methods

### Patients and Tissue

This study was approved by the Institutional Review Board and Ethics Committee of National Cheng Kung University Hospital. It included 16 men and 18 women and was conducted in accordance with the tenets of the Declaration of Helsinki.^12^ All surgeries were performed from September 1, 2021, to December 31, 2023, with pterygial excision and conjunctival autografting performed using fibrin glue. Specimens of pterygium and ipsilateral pterygium-free conjunctiva were taken from 34 patients during the surgical procedures. The tissue specimens were harvested from the superior bulbar area of the conjunctiva. None of the patients had any other ocular surface diseases, nor did they have a history of long-term topical eye drop use or an immunocompromised condition. The human tissue experiments complied with the guidelines of the ARVO Best Practices for Using Human Eye Tissue in Research.

### PEC Culture

Specimens of pterygium were cut into small pieces (around 1 mm^3^ in size) under a stereomicroscope, rinsed with phosphate-buffered saline (PBS), and placed in the wells of a six-well plate with the epithelium facing upward. PECs with similar shapes grew out from the edges of the specimens after 3 days. Fibroblast contamination was minimized by removing the tissue when a sufficient number of epithelial cells surrounded each explant. The epithelial cells were cultured up to three to seven passages before use. A purity of 98% was established using flow cytometry with cytokeratin antibodies. CECs from ipsilateral pterygium-free conjunctiva were cultured in the same manner as PECs.

### UVB Irradiation of Cultured Cells

Epithelial cells were seeded at approximately 5 × 10^5^ cells in a 60-mm culture dish (GeneDireX, Taoyuan, Taiwan) and grown in 10% fetal bovine serum–Dulbecco's Modified Eagle Medium. When cells reached semi-confluence, the medium was aspirated and cells were washed three times with PBS and left in a serum-free medium for 16 hours.^6^ This medium was replaced with PBS (2 mL), and cell monolayers were irradiated with UVB light (G15T8E; SANKYO, Tokyo, Japan) at 20 mJ/cm^2^. The UVB light intensity was monitored and calibrated before each experiment with the aid of a radiometer–photometer (Model 6.0; Solarmeter, Glenside, PA, USA). UVB exposure time (*t*) was determined using the equation *t* = UVB dose (mJ/cm^2^)/fluence rate (mW/cm^2^) as previously described.^13^ Some cells were incubated in PBS for an equivalent time without irradiation. UVB-irradiated cells were employed immediately upon exposure, and 600,000 cell lysates were administered to 200,000 PECs grown to 80% confluence in culture plates. Non-irradiated PECs lysed using a sonicator were used as controls as previously described.^7^^,^^10^

### Synthetic U1 RNA Oligonucleotide Treatment

PECs were treated for 24 hours with 1 µg of synthetic oligonucleotides representing different U1 small nuclear RNA (snRNA) structures: loop a sequence, 5′-GGGAGAACCAUGAUCACGAAGGUGGUUUUCCC-3′; loop b sequence, 5′-GGGCGAGGCUUAUCCAUUGCACUCCGGAUGUGCUCCCC-3′; loop c sequence, 5′-CGAUUUCCCCAAAUGUGGGAAACUCG-3′; and loop d sequence, 5′-UAGUCCCCCACUGCGUUCGCGCUUUCCCCUG-3′. These oligonucleotides correspond to the four major stem–loop structures in human U1 snRNA.^13^

### Cell Proliferation Assay and Treatment With Poly(I:C) and Cell Lysates

PECs and CECs were seeded at a density of 2 × 10^4^/100 µL in 96-well plates and cultured overnight. The medium was changed, and cells were then cultured in medium (control) or in medium containing various concentrations of poly(I:C). After treatment, water-soluble tetrazolium salt-1 (WST-1) reagent (630118; Clontech Laboratories, San Jose, CA, USA) was added, and cells were incubated for another 2 hours at 37°C. Absorbance was determined using a microplate reader at a test wavelength of 450 nm. In addition, PECs and CECs were treated with cell lysates from UVB-irradiated or non-irradiated PECs. Cell lysates, except for one group (UVB-irradiated cell lysate without Xfect polymer), were cotreated with Xfect polymer (631318; Clontech Laboratories) to enhance dsRNA entry.

### Western Blotting

After treatment, cells were lysed with a protein extraction solution containing protease inhibitors, and protein concentrations were measured using the bicinchoninic acid assay (Thermo Fisher Scientific Waltham, MA, USA), with bovine serum albumin as the standard. Then, 30 µg of crude protein was separated on 10% sodium dodecyl sulfate polyacrylamide gel electrophoresis gels and transferred to polyvinylidene difluoride membranes. The membranes were immunoblotted overnight at 4°C, with each primary antibody at the indicated dilution. The primary antibodies were obtained from Abcam (Cambridge, UK) and included TLR3 antibody (ab62566), NF-κB p65 antibody (ab32536), NF-κB (phosphorylated S536) antibody (ab76302), and glyceraldehyde 3-phosphate dehydrogenase (GAPDH) antibody (ab8245). The membranes were washed three times with Tris-buffered saline with Tween 20 (TBST) and exposed to horseradish peroxidase (HRP)-conjugated secondary antibodies for 1 hour at room temperature. Immunoreactive bands were detected using enhanced chemiluminescence with the Clarity Western ECL substrate (170-5060; Bio-Rad Laboratories, Hercules, CA, USA). The intensity of the bands was analyzed using an imaging system (BioSpectrum; UVP, Upland, CA, USA).

### Quantitative Real-Time PCR

Total RNA was extracted from the PECs and CECs with TRIzol reagent (Invitrogen, Carlsbad, CA, USA). A reverse transcription kit (FYT501; Yeastern Biotech, New Taipei City, Taiwan) was used for reverse transcription of total RNA (1 µg). Applied Biosystems SYBR Green Master Mix (Thermo Fisher Scientific) was used for the polymerase chain reaction (PCR) according to the instructions supplied with the StepOne real-time PCR system. The expression of each mRNA was calculated as expression relative to that of GAPDH mRNA. All data are presented as fold changes against each control. The gene primers were as follows: TLR3 forward, AGAGTTGTCATCGAATCAAATTAAAG; TLR3 reverse, AATCTTCCAATTGCGTGAAAA; TRIF forward, CAGGACGCCATAGACCACTC; TRIF reverse, TCCAGGTGTTGGCTCTGTTC; NF-κB forward, GGCTTCTATGAGGCTGAG; NF-κB reverse, GTTGTTGTTGGTCTGGATG; IL-6 forward, ACATCCTCGACGGCATCTCA; IL-6 reverse, CACCAGGCAAGTCTCCTCATT; IL-8 forward, CACTGCGCCAACACAGAAATTA; IL-8 reverse, GCTTGAAGTTTCACTGGCATCT; GAPDH forward, AGCCACATCGCTCAGACAC; and GAPDH reverse, GCCCAATACGACCAAATCC.

### RNA-Sequencing Analysis

Purified RNA was used to prepare the sequencing library with an Illumina TruSeq Stranded mRNA Library Prep Kit according to the manufacturer's instructions. Briefly, mRNA was purified from total RNA (1 µg) using oligo(dT)-coupled magnetic beads and fragmented into small pieces under elevated temperature. The first-strand complementary DNA (cDNA) was synthesized using reverse transcriptase and random primers. After the generation of double-stranded cDNA and adenylation on the 3′ ends of DNA fragments, the adaptors were ligated and purified with the AMPure XP system (Beckman Coulter, Brea, CA, USA). The quality of the libraries was assessed using the Agilent 2100 Bioanalyzer system and a real-time PCR system. The qualified libraries were then sequenced on an Illumina NovaSeq 6000 platform with 150 bp paired-end reads generated by a Genomics system (Genomics BioSci & Tech Co., Ltd., New Taipei City, Taiwan).

### Enzyme-Linked Immunosorbent Assay

The supernatants of cultured PECs were collected at 0, 6, 12, and 24 hours, and IL-6 and IL-8 concentrations were determined using enzyme-linked immunosorbent assay (ELISA) kits (BD Biosciences, Franklin Lakes, NJ, USA) according to the manufacturer's instructions. Assays were performed in triplicate and repeated three times.

### Statistical Analysis

All results are expressed as mean ± standard error of the mean (SEM). Student's *t-*test was used to analyze differences between groups, with *P* < 0.05 considered statistically significant. SigmaPlot (Systat Software, San Jose, CA, USA) was used for the analyses.

## Results

### U1 RNA Was Induced to Increase in Conjunctival and PECs by UVB Irradiation

**Figure 1.**
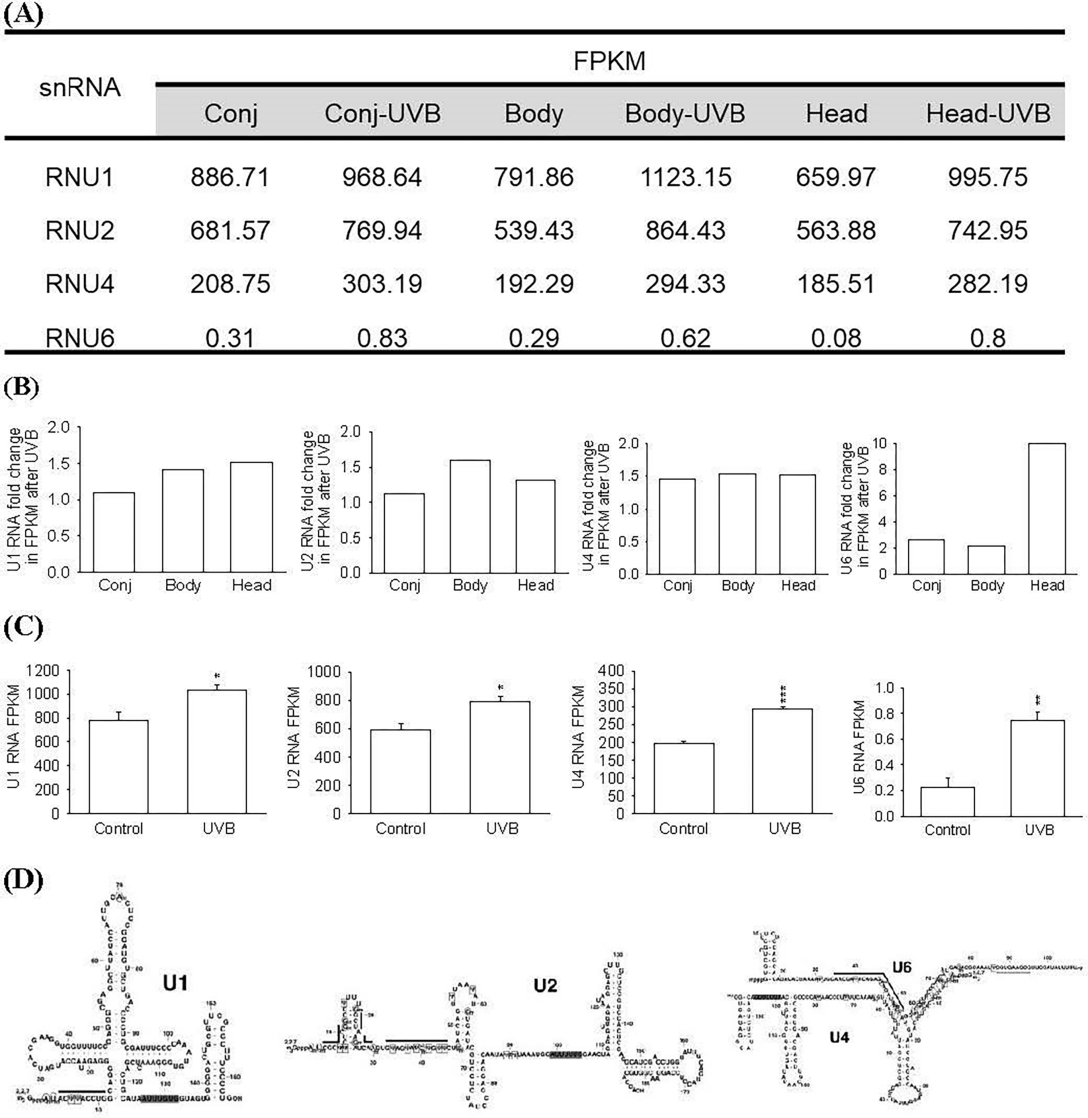
RNA-sequencing analysis of snRNAs in cultured CECs and PECs (head and body portions of pterygium). (**A**, **B**) FPKM and relative fold changes of U1, U2, U4, and U6 RNAs in cultured cells 24 hours after UVB irradiation (20 mJ/cm^2^) compared to cell groups without UVB exposure. (**C**) Average FPKM of U1, U2, U4, and U6 RNAs in cells cultured from three areas in the same eye (ipsilateral pterygium-free conjunctiva and the head and body portions of pterygium) (*n* = 3). *P < 0.05, **P < 0.01, ***P < 0.001 versus control. (**D**) Representation of bases and stem–loop structures of U1, U2, U4, and U6 RNA. Comparisons were made using Student's *t-*test. Data are presented as mean ± SEM. Conj, conjunctiva; body, body portion of pterygium; head, head portion of pterygium.

The base-reading frequency of snRNAs was compared between UVB-irradiated cells (CECs, from ipsilateral pterygium-free conjunctiva, and PECs, the head and body sections of pterygium) and non-irradiated cells (control group) (Figs. 1A, 1B). Fragments per kilobase of transcript per million mapped reads (FPKM) of U1, U2, and U4 were similar between CECs and PECs, with higher reads in CECs. The average FPKM of U1, U2, U4, and U6 RNAs was calculated for cells cultured from three areas in the same eye (ipsilateral pterygium-free conjunctiva and head and body portions of pterygium).

RNA-sequencing analysis revealed that UVB irradiation of cells led to an increase in the snRNAs (U1, U2, U4, and U6), with 1.32-fold, 1.33-fold, 1.5-fold, and 3.31-fold changes, respectively, in the average FPKM of conjunctiva and of head and body portions of pterygium compared to non-irradiated cells (*n* = 3; *P* < 0.05 for U1 and U2 RNA, and *P* < 0.01 for U4 and U6 RNA) (Figs. 1A, 1C). U1 RNA had the highest number of FPKM among these snRNAs, whereas U6 RNA had the lowest. In addition, UVB irradiation led to the highest stimulation of snRNAs in pterygial head and body compared to those in the conjunctiva (Fig. 1A).

### Differential Gene Expression Profile of CECs and PECs With or Without UVB Irradiation

**Figure 2.**
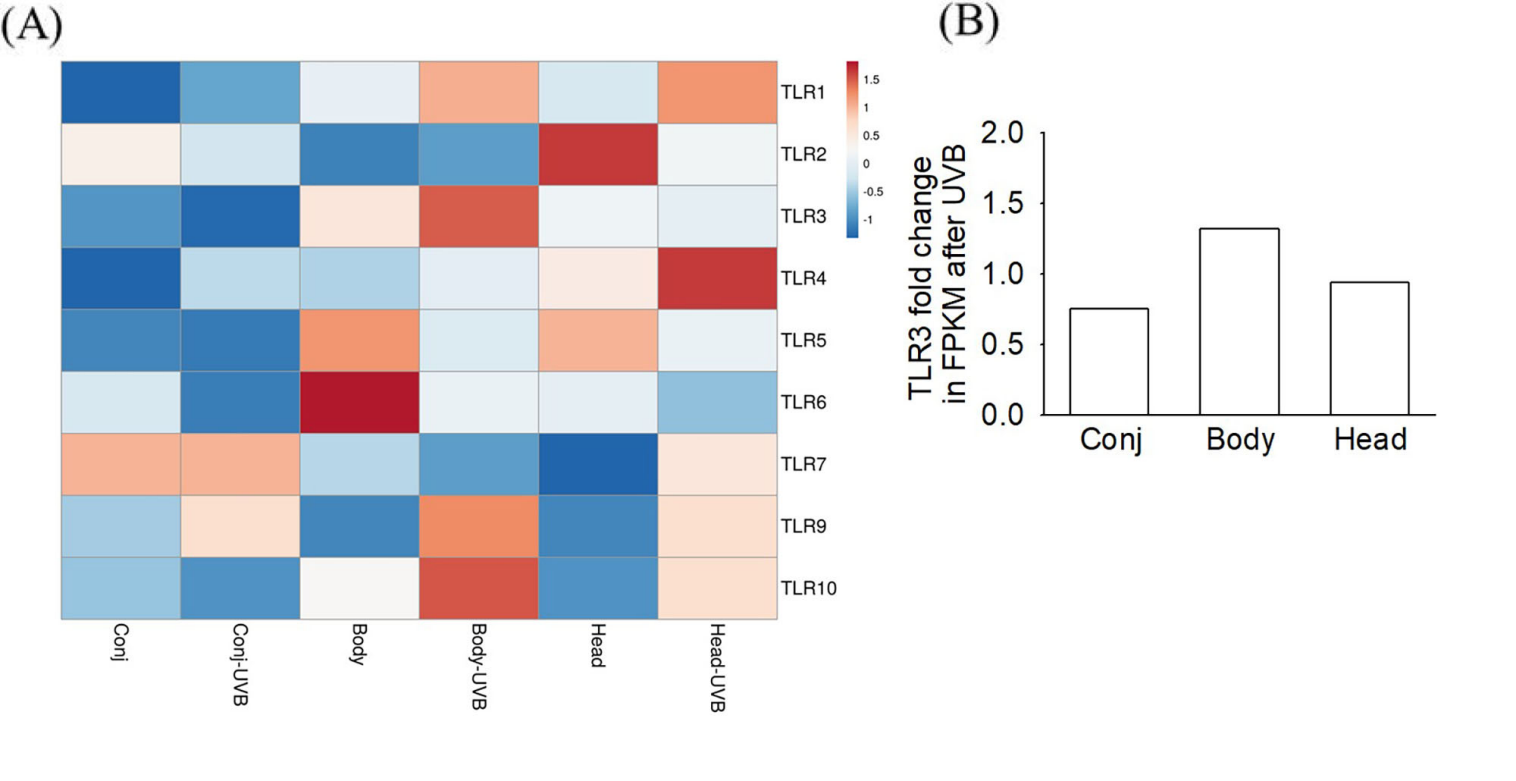
Differential expression of TLR family genes in CECs and PECs. (**A**) Heatmap of gene expression before and after UVB irradiation. (**B**) Fold change of TLR3 expression (FPKM) following UVB irradiation. Data were visualized using the ClustVis tool.

Using the RNA-sequencing data for differential gene expression analysis, expression of the genes of the TLR family was compared between CECs and PECs (body or head) with or without UVB irradiation (Fig. 2A). TLR1, TLR4, and TLR9 expression all increased after UVB irradiation of CECs and PECs. TLR3 expression was stimulated by UVB irradiation only in the pterygial body (1.32-fold), whereas it slightly decreased in the pterygial head (0.94-fold) and conjunctival cells (0.75-fold) (Fig. 2B).

In addition, genes differentially expressed in CECs and PECs were studied using Gene Set Enrichment Analysis (GSEA). Genes were screened according to the standard of FPKM > 0.3, 2-fold change, and *P* < 0.05 significance. In the RNA-sequencing results, cytokines including IL-1α and IL-8, features of senescence-associated secretory phenotypes, were increased after UVB irradiation (Supplementary Fig. S1A). Furthermore, p38 and p21 expression was stimulated, indicating a relationship between UVB irradiation and cellular senescence. Another pathway of significance was the spliceosome, which involves U1 RNA. The most enhanced gene of U1, U2, U4, and U6 RNA components was the *Sm* gene (Supplementary Fig. S1B), which encodes small nuclear ribonucleoprotein polypeptides B and B1. This protein is a component of U1, U2, U4/U6, and U5 small ribonucleoprotein particles (snRNPs) and helps pre-mRNA splicing or shaping of snRNP structure.^14^^–^^16^

### Synthetic U1 RNA-Induced Upregulation of TLR3 Signaling and Cytokine Production in PECs

**Figure 3.**
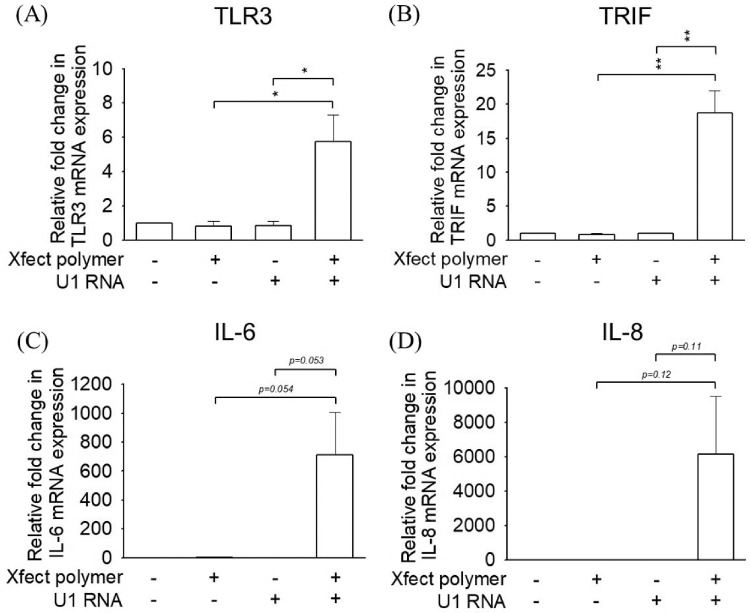
Quantitative PCR study of (**A**) TLR3, (**B**) TRIF, (**C**) IL-6, and (**D**) IL-8 in PECs after culture for 24 hours without treatment (control group) or with Xfect polymer only, synthetic U1 RNA (100 ng), or U1 RNA combined with Xfect polymer. Expression of TLR3, TRIF, IL-6, and IL-8 did not increase in PECs 24 hours after the addition of either Xfect polymer or synthetic U1 RNA compared to the control group, whereas only TLR3 and TRIF showed significant increases in PECs after the addition of synthetic U1 RNA combined with Xfect polymer (*n* = 4). *P < 0.05, **P < 0.01. Comparisons were made using Student's *t-*test. Data are presented as mean ± SEM.

The quantitative real-time PCR (qPCR) analysis revealed a significant increase in TLR3 and TRIF expression in PECs 24 hours after the addition of synthetic U1 RNA and Xfect polymer (*n* = 3, *P* < 0.05) (Figs. 3A, 3B). IL-6 and IL-8 production was likewise stimulated in PECs by synthetic U1 RNA and Xfect polymer (Figs. 3C, 3D). Moreover, TLR3, TRIF, IL-6, and IL-8 expression in PECs did not increase 24 hours after the addition of either Xfect polymer or synthetic U1 RNA.

### Poly(I:C)-Induced TLR3 Signaling, Cytokine Production, and Cell Proliferation in PECs and CECs

**Figure 4.**
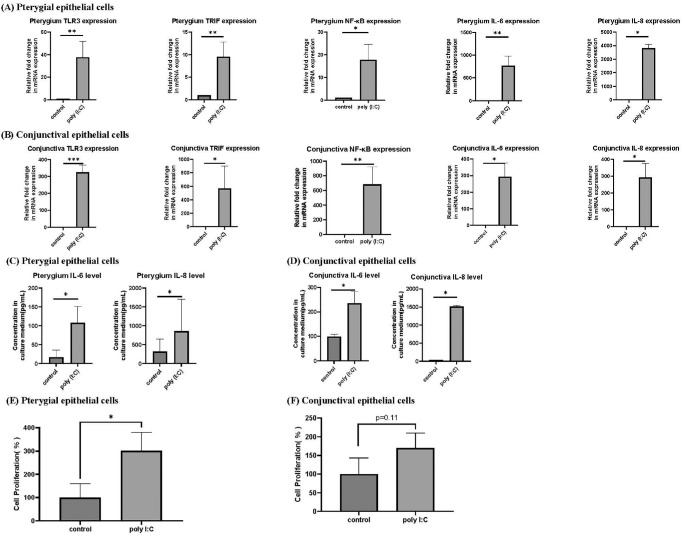
(**A**, **B**) Quantitative PCR study of TLR3, TRIF, NF-κB, IL-6, and IL-8 in PECs after culture for 24 hours without any treatment (control group) or with poly(I:C) (10 µg/mL). The expression of TLR3, TRIF, NF-κB, IL-6, and IL-8 increased in PECs and CECs 24 hours after the addition of poly(I:C) compared to the control group (*n* = 3). *P < 0.05, **P < 0.01, ***P < 0.001. (**C**, **D**) ELISA analysis of IL-6 and IL-8 in PECs and CECs after culture for 24 hours without any treatment and with poly(I:C) (10 µg/mL). Poly(I:C) induced IL-6 and IL-8 secretion in both CECs and PECs (*n* = 3). (**E**, **F**) WST-1 assay of CEC and PEC proliferation after poly(I:C) treatment (*n* = 3). Comparisons were made using Student's *t-*test. Data are presented as mean ± SEM.

The qPCR and ELISA studies were performed to evaluate the influence of poly(I:C) on the TLR3 pathway in CECs and PECs. The qPCR results revealed that 10 µg/mL of poly(I:C) significantly induced TLR3, TRIF, NF-κB, IL-6, and IL-8 expression in PECs and CECs compared to the control group (Figs. 4A, 4B). ELISA experiments also showed that poly(I:C) increased IL-6 and IL-8 secretion in PECs and CECs (Figs. 4C, 4D). The WST-1 cell proliferation assay showed that poly(I:C) increased cell proliferation in both CECs and PECs (*P* = 0.11 for CECs and *P* < 0.05 for PECs) (Figs. 4E, 4F).

### UVB-Irradiated PEC Lysates Induced TLR3 Signaling, Cytokine Production, and PEC and CEC Proliferation

**Figure 5.**
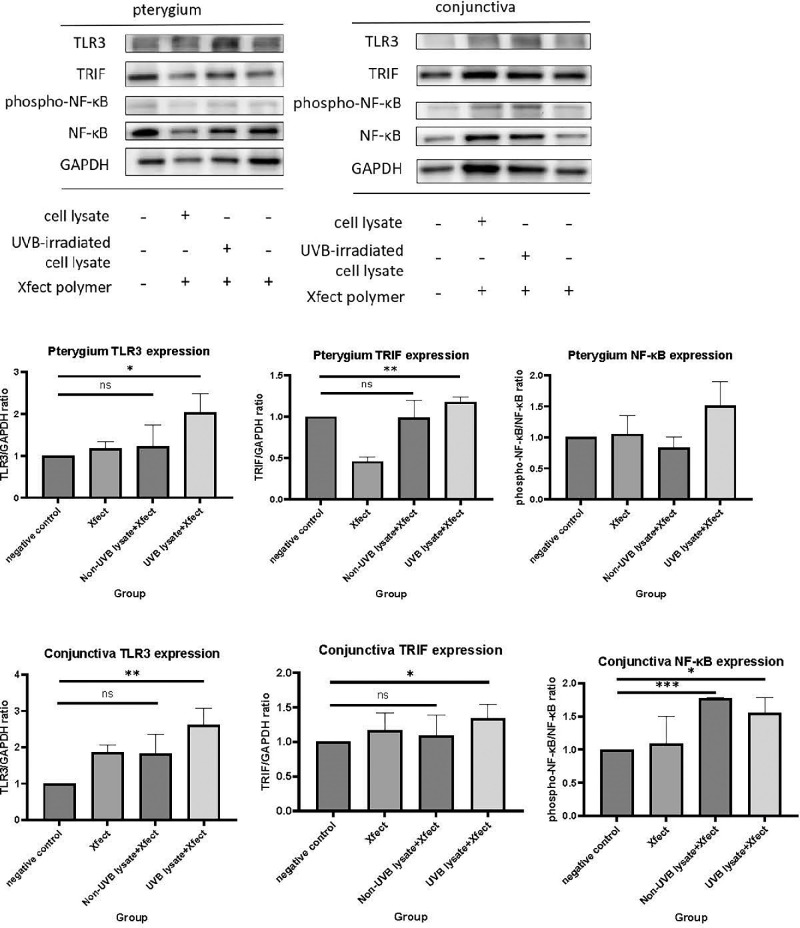
Western blot analysis of TLR3, TRIF, and NF-κB in CECs and PECs after 24 hours without treatment (control group) or the addition of Xfect polymer, non UVB-irradiated PEC lysates, or UVB-irradiated PEC lysates (with Xfect polymer). TLR3 and TRIF were enhanced in both CECs and PECs by UVB-irradiated PEC lysates. Comparisons were made using Student's *t-*test. Data are presented as mean ± SEM (n = 3). *P < 0.05, **P < 0.01, ***P < 0.001.

**Figure 6.**
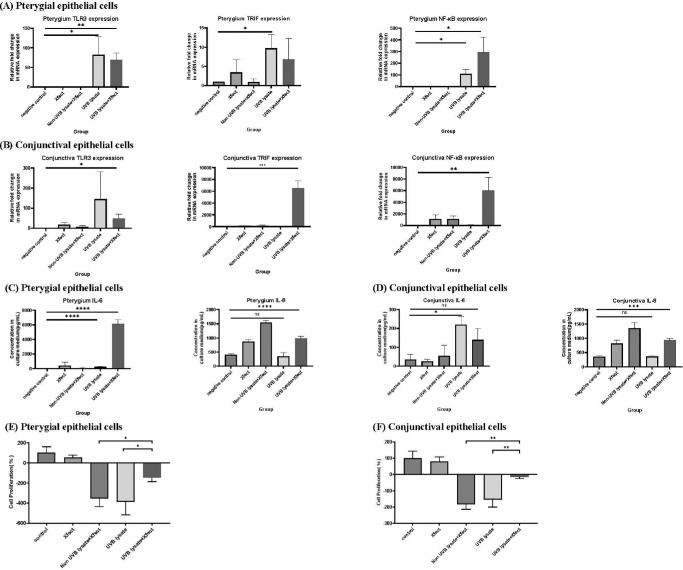
(**A**, **B**) Quantitative PCR study of TLR3, TRIF, and NF-κB in conjunctival epithelial cells (CECs) and pterygial epithelial cells (PECs) after culture for 24 hours without treatment or addition of Xfect polymer, non UVB-irradiated PEC lysates, or UVB-irradiated PEC lysates (with or without Xfect polymer). Expression of TLR3 and NF-κB significantly increased in CECs and PECs after the addition of UVB-irradiated PEC lysates compared to the control group (*n* = 3). (**C**, **D**) ELISA analysis of IL-6 and IL-8 in CECs and PECs. IL-6 levels were higher after the addition of UVB-irradiated PEC lysates compared to the levels in other groups. In addition, IL-8 was elevated in CECs and PECs of the UVB-irradiated PEC lysates group (*n* = 3 for CECs and *n* = 6 for PECs). *P < 0.05, **P < 0.01, ***P < 0.001, ****P < 0.0001. (**E**, **F**) WST-1 assay of CEC and PEC proliferation after PEC lysate treatment. PEC lysates cotreated with Xfect polymer enhanced cell proliferation compared to the group treated with cell lysates only (with or without UVB irradiation). Comparisons were made using Student's *t-*test. Data are presented as mean ± SEM.

After confirming TLR3 activation in CECs and PECs by poly(I:C), we further investigated the effects of UVB-irradiated PEC lysates on TLR3 signaling. PECs were irradiated with 20 mJ/cm^2^ UVB and lysed. Then, whole cell lysates (600,000-cell equivalent) transfected with Xfect polymer were administered to 200,000 PECs. Our prepared cell lysates mimicked UV-damaged cell debris on the ocular surface and contained segmented RNA. Results of western blot showed that UVB-irradiated PEC lysates significantly enhanced TLR3 and TRIF expression in PECs (Fig. 5). Similarly, TLR3, TRIF, and NF-κB mRNA expression was significantly induced after treatment with UVB-irradiated PEC lysates (Figs. 6A, 6B). TLR3 expression induced by UVB-irradiated PEC lysates was similar in CECs and PECs, but TRIF and NF-κB expression was more strongly induced in CECs than in PECs. IL-6 and IL-8 secretion after treatment with UVB-irradiated PEC lysates significantly increased in both CECs and PECs (Figs. 6C, 6D). Cell proliferation after treatment with UVB-irradiated PEC lysates was studied using the WST-1 cell proliferation assay (Figs. 6E, 6F). Results showed that the rate of proliferation of CECs and PECs was reduced in groups treated with UVB-irradiated PEC lysates compared to the control group. However, co-treatment with Xfect polymer induced cell growth compared to the lysate-treated groups (*P* < 0.05 for CECs).

### RNase A Decreased TLR3 Signaling in UVB-Irradiated PECs

**Figure 7.**
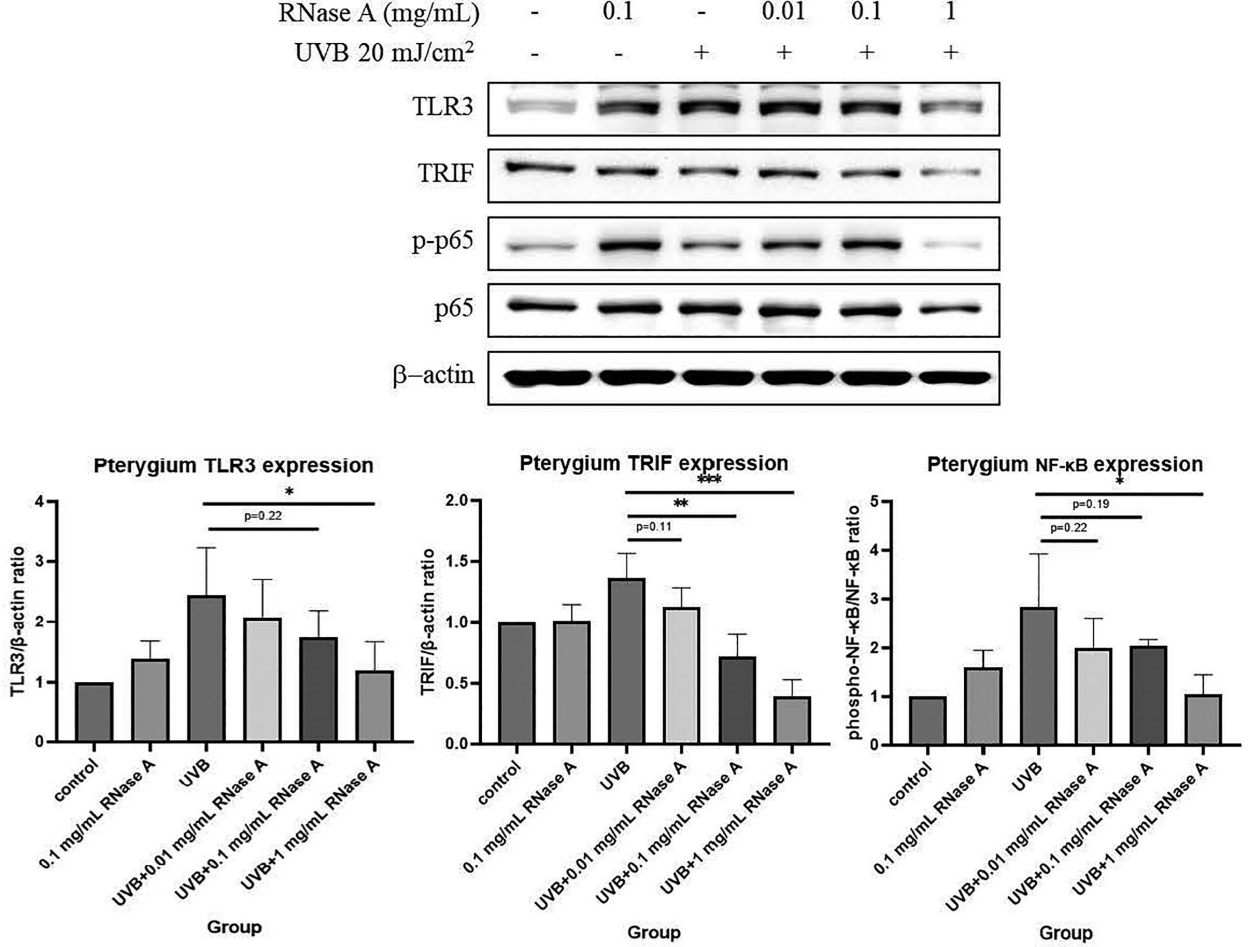
Western blot analysis of TLR3, TRIF, and NF-κB in cultured PECs pretreated with various concentrations (0.01, 0.1, and 1 mg/mL) of RNase A and receiving UVB irradiation (20 mJ/cm^2^). After culture for 24 hours, PECs that were treated with 1 mg/mL RNase A showed a significant decrease in TLR3, TRIF, and phospho-NF-κB expression (*n* = 3). *P < 0.05, **P < 0.01. Comparisons were made using Student's *t-*test. Data are presented as mean ± SEM.

We previously observed that RNA released from PEC lysates and poly(I:C) could induce TLR3 signaling in CECs and PECs. Therefore, we used western blot to assess the inhibitory effect of RNase A on TLR3 signaling after UVB irradiation. TLR3, TRIF, and the phospho-NF-κB/NF-κB ratio significantly increased after UVB irradiation, and RNase A treatment decreased their levels in a dose–response manner, indicating that RNA played an important role in the activation of TLR3 signaling in pterygium (Fig. 7).

## Discussion

In previous studies, UVB irradiation was found to induce IL-6 and IL-8 in cultivated human PECs and limbal epithelial cells^6^ and upregulate IL-6 and TNF-α in human corneal epithelial cells.^17^^,^^18^ Pterygium formation may be influenced by IL-6 and IL-8, which promote chronic inflammation, cell proliferation, and invasive capabilities.^19^^,^^20^ IL-8 also promotes angiogenesis, which is involved in primary and secondary ocular inflammation.^21^^–^^23^ Consistent with the findings of previous studies, our qPCR and ELISA results showed that poly(I:C) induced IL-6 and IL-8 production in both CECs and PECs. IL-6 and IL-8 are both downstream products of the TLR3 signaling pathway, which has been studied in poly(I:C)-stimulated human CECs,^24^ corneal epithelial cells and fibroblasts,^20^^,^^25^ intestinal epithelial cells,^26^ and aortic valve interstitial cells.^27^

The TLR3 signaling pathway was found to be activated in UVB-irradiated PECs in our previous study.^5^ Thus, we investigated TLR3 and TRIF expression after poly(I:C) stimulation. Results indicated that poly(I:C) increased the mRNA expression of TLR3 and TRIF in PECs or CECs. In addition, WST-1 assay results indicated that poly(I:C) increased the proliferation of CECs and PECs. In poly(I:C)-treated human corneal epithelial cells, TLR3 activation has been found to be linked to the production of NF-κB^20^ and IL-6 and IL-8.^25^ We observed similar trends after poly(I:C) treatment of CECs and PECs, which further consolidated the TLR3 and NF-κB signaling pathway in epithelial cells of the ocular surface.

UV rays chronically damage epithelial cells and cause necrosis and cell death.^28^^,^^29^ Intracellular contents, identified as DAMPs, are discharged into extracellular spaces, resulting in inflammation, including the release of IL-6 and IL-8. Previous studies have shown that RNA released from injured keratinocytes is capable of activating TLR3 and triggering inflammation.^10^ In the present study, we discovered that lysates of UVB-irradiated PECs induced TLR3, TRIF, NF-κB, IL-6, and IL-8 upregulation in CECs and PECs through activation of the TLR3 signaling pathway. PECs and Xfect polymer (used for transfecting RNA in cell lysates) not irradiated with UVB were also tested for their effects on TLR3 signaling, but significant upregulation of TLR3, TRIF, and NF-κB was rarely observed. The WST-1 assay indicated an increase in cell proliferation in groups treated with UVB-irradiated cell lysates and Xfect polymer. Interestingly, cell proliferation was mostly reduced in groups treated with only cell lysates, which indicates that there might be unknown harmful components in lysates that promote cell death. Because RNA released from PECs might activate TLR3 signaling in CECs or PECs, we evaluated the inhibitory effect of RNase A on TLR3 after UVB irradiation. Results showed that RNase A reduced TLR3, TRIF, and phospho-NF-κB in PECs, which confirmed that RNA from UVB-damaged PECs was necessary for activating the TLR3/NF-κB pathway in pterygium.

Non-coding RNAs can influence a wide array of biological processes in humans.^30^ Previous studies showed that U1 RNA, a type of snRNA linked to innate immune signaling, was the most abundant ncRNA in UVB-irradiated human epidermal keratinocytes (HEKs).^10^ UVB fragmented U1 RNA into segments shorter than 100 bp; these fragments elevated IL-6 and TNF-α production in nonirradiated HEKs.^10^ Based on these findings, we hypothesized that different types of non-coding RNAs might be involved in the etiology of pterygium. U1, U2, U4, and U6 RNA levels increased in UVB-irradiated CECs and PECs in our investigation. Among these snRNAs, U1 RNA was the most prevalent. Furthermore, UVB stimulated the highest snRNA production in pterygial head and body compared to the conjunctiva.

Consistent with the results of our cellular experiments, RNA-sequencing analysis revealed an increase in TLR3, IL-6, and IL-1β expression in PECs and CECs irradiated with UVB compared to their respective controls. In addition, components of U1, U2, U4/U6, and U5 RNA were also induced after UVB irradiation, indicating their importance in the pathogenesis of pterygium. The expression of TLR family genes was detected in conjunctival and pterygial samples. TLR3, TLR7, and TLR9 increased after UVB irradiation, which may indicate RNA and DNA sensing after UVB damage to CECs and PECs. TLR 9 has been reported to be overexpressed by CECs in response to microbial keratitis, especially *Pseudomonas aeruginosa* keratitis.^31^^,^^32^ Both TLR7 and TLR9 have been reported to be overexpressed in herpes simplex keratitis.^33^ Interestingly, TLR3 expression was elevated by UVB irradiation in the pterygial body, but not the pterygial head and conjunctiva. These results correspond to those of our previous study on human pterygial and conjunctival tissue, which showed that TLR3 expression was higher in the pterygial body.^5^ p63, a downstream effector of TLR3 signaling, was highly expressed in the pterygial head, which exhibited rapid proliferation into the cornea.^5^

Endogenous RNA released after UVB irradiation is capable of activating the TLR3 signaling pathway.^10^ UVB irradiation damages normal human skin epidermal keratinocytes, resulting in the production of U1 RNA containing a secondary structure composed of double-stranded stem–loops. U1 RNA was found to activate TLR3 and induce translocation of the NF-κB subunit p65.^10^ Consistent with this, we observed a significant rise in TLR3, TRIF, IL-6, and IL-8 expression in PECs in the group cotreated with synthetic U1 RNA and Xfect polymer. These findings suggest that U1 RNA may operate as a type of DAMP in PECs, activating the TLR3 signaling pathway.

TLR3 activation results in the generation of pro-inflammatory mediators such as macrophage inflammatory protein-1 (MIP-1), IL-6, IL-8, RANTES, and TNF-α.^8^^,^^20^ UVB irradiation has been shown to induce IL-6 and IL-8 in cultured PECs and limbal epithelial cells in a time- and dose-dependent manner. By increasing angiogenesis, chronic inflammation, cell proliferation, and invasive capabilities, IL-6 and IL-8 may contribute to pterygium formation.^6^ Echoing previous studies, both UVB-irradiated cell lysates and synthesized U1 RNA were able to promote IL-6 and IL-8 production in PECs in our investigation. Therefore, we inferred that UVB radiation might induce necrosis in PECs and the release of specific RNAs with stem–loops, such as U1 RNA. The stem–loops of U1 RNA may activate the TLR3 signaling pathway and generate IL-6 and IL-8.

**Figure 8.**
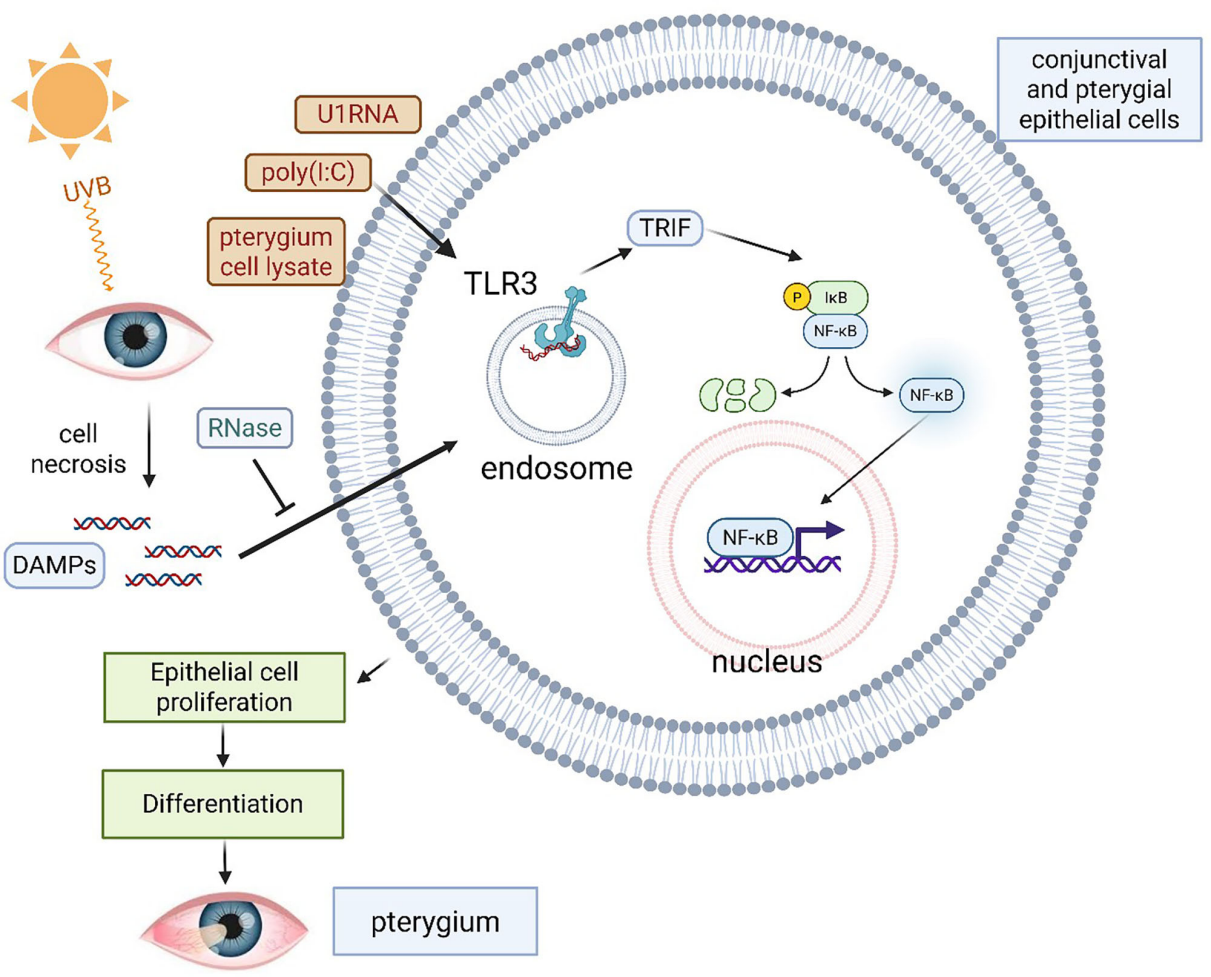
Graphical summary of the signaling mechanisms investigated in this study. Damaged RNA molecules from the ocular surface activate TLR3 signaling and promote pterygium formation.

In conclusion, poly(I:C) and UVB-irradiated PEC lysates activated the TLR3 signaling pathway, resulting in increased IL-6 and IL-8 secretion and enhanced cell proliferation in PECs. UVB irradiation elevated endogenous noncoding U1 RNA in these cells, and synthetic U1 RNA further activated TLR3 signaling. These findings suggest that snRNA may play a critical role in the UV-related pathogenesis of pterygium, as TLR3 recognizes U1 RNA as a DAMP released from UV-damaged necrotic cells (Fig. 8). The consequent overproduction of IL-6 and IL-8 likely promotes PEC proliferation.

A limitation of this study is that the cellular origin of pterygium could not be determined using only PECs and CECs. Future research should examine the relationship between proliferative activity and TLR3 expression in other potential sources of pterygium, including fibroblasts^34^ and limbal stem cells.^35^ Furthermore, the therapeutic potential of targeting TLR3 warrants investigation, such as with TLR3 inhibitors (e.g., CU-CPT4a) and antiproliferative agents (e.g., cyclosporine A).

## Supplementary Material

Supplement 1
