# Supplementary material for: U1 RNA Detected by Toll-Like Receptor 3 Plays a Role in the Pathogenesis of Pterygium
Source: Invest Ophthalmol Vis Sci. 2025 Dec 3;66(15):15. doi: 10.1167/iovs.66.15.15 (PMC12697701; doi:10.1167/iovs.66.15.15)
Supplement: Supplement 1 [file iovs-66-15-15_s001.docx]

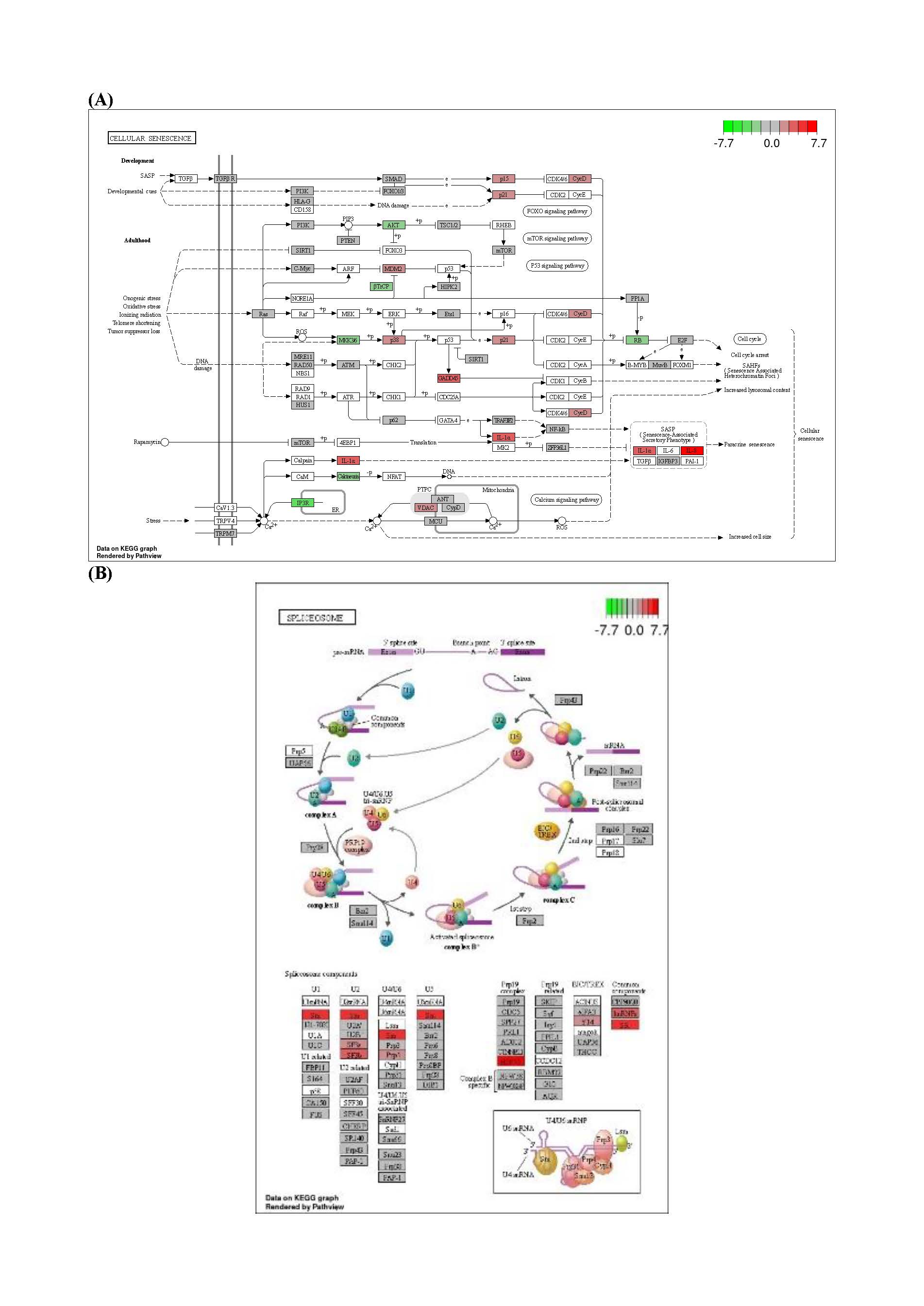


**Supplementary Figure 1.** (A) Differences in gene expression of the cellular senescence pathway (KEGG) between UVB-irradiated and control pterygial epithelial cells. (B) Differences in gene expression of the spliceosome pathway (KEGG) between UVB-irradiated and control pterygial epithelial cells.
